# A Seal That Heals: Resident & Staff Perspectives on PARO in a Long-Term Care Home

**DOI:** 10.1093/geroni/igaf122.3692

**Published:** 2025-12-31

**Authors:** Yuka Ohno, Janna Zeid, Lillian Hung

**Affiliations:** University of British Columbia, Vancouver, British Columbia, Canada; University of British Columbia, Vancouver, British Columbia, Canada; University of British Columbia, Vancouver, British Columbia, Canada

## Abstract

In Canada, dementia is highly prevalent in long-term care (LTC), with estimates suggesting that nearly two-thirds of residents live with some form of cognitive impairment. Within LTC, residents often face social isolation, loneliness, and agitation, challenges compounded by limited staffing resources. Socially assistive robots, such as PARO, have been developed to reduce stress and promote well-being. Yet, most research has examined either LTC residents’ or staff’s perspectives in isolation, limiting understanding of its broader impact. Our study explored the experiences of both residents with dementia and staff when using PARO in an LTC setting. Over four weeks from February to March 2025, residents (n = 10) engaged with PARO during group session, while staff (n = 10) participated in reflective sessions informed by video excerpts of these interactions. Reflexive thematic analysis revealed three themes from residents’ and staff’s perspectives respectively. For residents, PARO brought delight, fostered emotional validation, and promoted social connections and shared experiences. Staff feedback corroborated those themes, adding that PARO was a potential resource in the care toolkit to support person-centered care, and that its unique features generated strong engagement, but also presented physical accessibility, feasibility, and sustainability challenges. Findings highlight that PARO can be a valuable tool to enhance dementia care when both resident and staff perspectives are considered. Sustainable adoption requires collaboration among staff, administrators, and researchers to address accessibility, integration, and long-term feasibility. This study contributes to a holistic understanding of PARO’s potential role in LTC and informs strategies for advancing socially assistive technologies in dementia care.

